# Artificial intelligence to predict bed bath time in Intensive Care Units

**DOI:** 10.1590/0034-7167-2023-0201

**Published:** 2024-02-26

**Authors:** Luana Vieira Toledo, Leonardo Lopes Bhering, Flávia Falci Ercole

**Affiliations:** I Universidade Federal de Viçosa. Viçosa, Minas Gerais, Brazil; II Universidade Federal de Minas Gerais. Belo Horizonte, Minas Gerais, Brazil

**Keywords:** Nursing, Baths, Artificial Intelligence, Neural Networks, Computer, Intensive Care Units, Enfermería, Baños, Inteligencia Artificial, Redes Neurales de la Computación, Unidades de Cuidados Intensivos, Enfermagem, Banhos, Inteligência Artificial, Redes Neurais de Computação, Unidades de Terapia Intensiva

## Abstract

**Objectives:**

to assess the predictive performance of different artificial intelligence algorithms to estimate bed bath execution time in critically ill patients.

**Methods:**

a methodological study, which used artificial intelligence algorithms to predict bed bath time in critically ill patients. The results of multiple regression models, multilayer perceptron neural networks and radial basis function, decision tree and random forest were analyzed.

**Results:**

among the models assessed, the neural network model with a radial basis function, containing 13 neurons in the hidden layer, presented the best predictive performance to estimate the bed bath execution time. In data validation, the squared correlation between the predicted values and the original values was 62.3%.

**Conclusions:**

the neural network model with radial basis function showed better predictive performance to estimate bed bath execution time in critically ill patients.

## RESUMO

**Objetivos:**

avaliar a *performance* preditiva de diferentes algoritmos de inteligência artificial para estimar o tempo de execução do banho no leito em pacientes críticos.

**Métodos:**

estudo metodológico, que utilizou algoritmos de inteligência artificial para predizer o tempo de banho no leito em pacientes críticos. Foram analisados os resultados dos modelos de regressão múltipla, redes neurais *perceptron* multicamadas e função de base radial, árvore de decisão e *random forest*.

**Resultados:**

entre os modelos avaliados, o modelo de rede neural com função de base radial, contendo 13 neurônios na camada oculta, apresentou melhor *performance* preditiva para estimar o tempo de execução do banho no leito. Na validação dos dados, o quadrado da correlação entre os valores preditos e os valores originais foi de 62,3%.

**Conclusões:**

o modelo de rede neural com função de base radial apresentou melhor *performance* preditiva para estimar o tempo de execução do banho no leito em pacientes críticos.

## RESUMEN

**Objetivos:**

evaluar el rendimiento predictivo de diferentes algoritmos de inteligencia artificial para estimar el tiempo de ejecución del baño en cama en pacientes críticos.

**Métodos:**

estudio metodológico, que utilizó algoritmos de inteligencia artificial para predecir el tiempo de baño en cama en pacientes críticos. Se analizaron los resultados de modelos de regresión múltiple, redes neuronales perceptrón multicapa y función de base radial, árbol de decisión y *random forest*.

**Resultados:**

entre los modelos evaluados, el modelo de red neuronal con función de base radial, que contiene 13 neuronas en la capa oculta, presentó el mejor desempeño predictivo para estimar el tiempo de ejecución del baño en cama. En la validación de datos, la correlación al cuadrado entre los valores predichos y los valores originales fue del 62,3%.

**Conclusiones:**

el modelo de red neuronal con función de base radial mostró mejor rendimiento predictivo para estimar el tiempo de ejecución del baño en cama en pacientes críticos.

## INTRODUCTION

Artificial intelligence (AI) is a branch of computer science combined with cognitive science, in which computational systems are developed to carry out tasks that would require human intelligence. To this end, different techniques and models are used, highlighting machine learning^(1)^.

Using AI tools has been boosted by the need to obtain better results for patients at lower costs from more data^(2-3)^. Prediction methods based on multiple logistic or linear regressions have been used in different research; however, machine learning models offer the additional possibility of improving prediction based on detecting patterns of many variables simultaneously^(4)^. It is believed that, with advances in scientific technical evolution, AI will fundamentally transform healthcare and nursing care.

For nursing, AI tools offer great promise for optimizing care, while enabling decision-making and carrying out nursing interventions to be guided by the applicable use of data, information and knowledge, providing greater assertiveness^(5)^. Promising results have been obtained in the prediction of pressure injuries^(6)^ and early Intensive Care Unit (ICU) readmissions^(7)^.

Furthermore, there is an important role played by AI in the management of materials and human resources for patient care^(4)^. This applicability can be useful for sizing nursing staff, especially in units with high demand for care, such as ICUs, where work overload is a complaint frequently reported by the team and can cause greater absenteeism^(8)^.

Considering the activities performed by the nursing team in ICUs, it is clear that bed bath is one of the routinely performed interventions that requires scientific knowledge and technical skill from professionals^(9)^. It is not free from risks^(10)^, and has a significant influence on nursing team workload^(8)^. Studies show that bed bath can be influenced by individual characteristics, especially in relation to invasive device use as well as aspects linked to infrastructure and human and material resources^(8,10-11)^.

In this context, considering that multiple baths can be performed by the same professional during their working day and that execution time can vary according to patient characteristics, the relevance of studies that assess the topic is evident, especially aimed at building models for predicting bath time. It is believed that an accurate model can be used to build a useful management tool to instrumentalize nursing professionals in planning and executing care, in order to make the division of labor more equitable, minimizing risks for those who perform and those who receive. The work process in ICUs can be facilitated by being able to predict in advance how much time will be required by each patient during their bath, considering the influence of their clinical condition. It is noteworthy that, to date, there are no studies using AI algorithms to predict bed bath time in critical care, thus justifying the innovative nature of this study.

## OBJECTIVES

To assess the predictive performance of different AI algorithms to estimate bed bath execution time in critically ill patients.

## METHODS

### Ethical aspects

The study was conducted in accordance with national and international ethical guidelines regarding research with human beings, and was approved by the Research Ethics Committee of the proposing institution.

The Informed Consent Form was obtained from all family members of critically ill patients involved in the study by signing at the time of ICU admission.

### Study design, period and place

This is a methodological study that used AI algorithms to predict the time required to perform bed baths in critically ill patients. Bath time prediction was carried out based on clinical variables collected in a randomized clinical trial^(12)^.

The data was analyzed by AI algorithms from June to October 2022. The aforementioned clinical study that gave rise to the data set was carried out in an ICU, which has six beds dedicated to the care of critical patients arising from clinical and surgical. The care team is made up of nurses, doctors, nursing technicians, physiotherapists and nutritionists.

### Sample; inclusion and exclusion criteria

In this study, information from all 50 adult critical patients who were admitted to the ICU and received the bed bath intervention during the clinical study was used.

### Study protocol

In the clinical study, patients were admitted to the ICU and received a bed bath using the traditional method, using a basin with soap and water. The bath was carried out by two nurses uninterruptedly, in the head-foot direction, with no oral hygiene performed. During the bath, a third researcher assessed patients’ sociodemographic and clinical variables and recorded the procedure execution time.

The data set used for analyzing and building predictive models included bed bath time (in minutes), considered as an outcome, in addition to the predictor variables: age (in years); sex (male/female); comorbidities (yes/no); use of vasoactive drugs (yes/no); sedatives (yes/no); oxygen therapy (yes/no); orotracheal tube/tracheostomy (yes/no); central venous catheter (yes/no); peripheral venous catheter (no/yes); indwelling bladder catheter (yes/no); nasoenteric/nasogastric catheter (yes/no); hemodynamic monitoring devices (yes/no); drains (yes/no); and ostomies (yes/no). Data related to bed bath time and patient characteristics were collected by another researcher who did not participate in the intervention.

### Analysis of results, and statistics

Data were analyzed using Rbio^®^ software version 170^(13)^. A descriptive data analysis was carried out, presenting absolute and relative frequencies of categorical variables and mean and standard deviation of continuous variables. To predict bed bath execution time, the results obtained by multiple regression, multilayer perceptron neural networks and radial basis function, decision tree and random forest were assessed.

Initially, the data was divided into 70% (n = 35), for training the algorithms, and 30% (n = 15), for validating the predictive performance of the adjusted models (test), being repeated 10 times. The square of the correlation of predicted data and original validation data was summarized by the value of the coefficient of determination R^2^. With the exception of multiple regression, for assessing the other models, the Root Mean Square Error (RMSE) and Mean Absolute Error (MAE) values were also used.

RMSE calculates the magnitude of the mean error by the square root of the mean of the squares of the errors. In this way, it assigns greater weight to errors of greater magnitude and lesser weight to errors of smaller magnitude. It is obtained in the same unit as the variable under analysis^(14)^.

MAE calculates the mean of the absolute differences between the predicted value and the actual value. MAE does not take into account whether the error is positive or negative, and absolute differences are not given weight^(14)^.

In multiple regression, the function that relates the predictors to the response of interest is restricted to linear forms. In neural networks, nonlinear transformations are applied to linear combinations of predictors, giving rise to latent units^(15)^. Networks have the ability to store knowledge based on the data used for training and, later, reproduce them according to the objective of the analysis^(16)^. In this study, multilayer perceptron neural networks were used with two types of training algorithms, such as resilient backpropagtion with weight backpropagation (perceptron 1) and without weight backpropagation (perceptron 2). In both models, the logistic activation function was used, and possible combinations of up to two hidden layers with up to 20 neurons each were tested. The neural network with a radial basis function comprises only one hidden layer, with a radially symmetric (Gaussian) activation function. In this model, the hidden layer was tested with up to 20 neurons. The objective decision tree combines predictions from a set of classifiers with an error rate slightly lower than that of a random classification, generating a decision tree with few divisions. In this study, the tree used contained up to six nodes. The last method assessed was random forest, in which 500 trees were sampled to obtain the results.

## RESULTS

The mean age of the patients who received the bed bath and served as the basis for building AI algorithms was 68.6 years (±19.0 years). There was a predominance of male patients 28 (56.0%) and 42 patients with comorbidities (84.0%), as shown in Table 1.

**Table 1:** Characteristics of critically ill patients who received a bed bath and served as a basis for building artificial intelligence algorithms (N=50)

Variables	Parameters
Age m (±sd)	68.6 (±19.0)
Sex n (%)	
Male	28 (56.0)
Female	22 (44.0)
Comorbidities n (%)	
No	8 (16.0)
Yes	42 (84.0)
Medications n (%)	
Sedatives	10 (20.0)
Vasoactive drugs	11 (22.0)
Devices n (%)	
Continuous infusion pump	47 (94.0)
Nasoenteric Catheter	19 (38.0)
Central Venous Catheter	8 (16.0)
Peripheral Venous Catheter	45 (90.0)
Indwelling Bladder Catheter	27 (54.0)
Drains	6 (12.0)
Invasive hemodynamic monitoring	4 (8.0)
Oxygen therapy	28 (56.0)
Ostomies	2 (4.0)
Endotracheal tube	12 (24.0)

During the clinical study, the mean bed bath time was 26.45 minutes (95% CI 25.07 - 27.82). From the analysis of AI algorithm metrics, it was evident that the neural network model with a radial basis function containing 13 neurons in the hidden layer showed the highest correlation between the predicted values and the original values (R^2^= 62.3%; RMSE = 0.7 and MAE =1.9). It is noteworthy that perceptron neural network 1 can also be considered an option for predicting bathing time, considering that it presented lower RMSE (0.5) and lower MAE (1.4), demonstrating that the predictions drawn by this algorithm are close to the real values (Table 2).

**Table 2:** Result of analysis of artificial intelligence algorithms to predict bed bath execution time

Method	R^2^ (%)	RMSE	MAE
Multiple regression	23.5	-	-
Perceptron neural network 1	55.3	0.5	1.4
Perceptron neural network 2	44.5	0.9	2.5
Neural network with radial basis function	62.3	0.7	1.9
Decision tree	7.4	4.7	4.1
Random forest	20.3	4.0	3.7

*R^2^ - Square of the correlation of predicted data with original data; RMSE - Root Mean Square Error; MAE - Mean Absolute Error.*

## DISCUSSION

The good performance of the predictive performance of the AI analyzes found in this study, especially the neural network with a radial basis function, can be considered promising for technical-scientific advancement in nursing. The use of these algorithms allows data to be analyzed in a more robust and efficient way than traditional statistical methods based on regression models, which allows for greater assertiveness^(17)^. AI algorithms offer the additional possibility of improving outcome prediction based on detecting patterns across many variables simultaneously^(4)^.

In the health sector, its use can be carried out with a view to improving treatment and results, promoting the efficiency and effectiveness of care processes as well as assessing patients’ behavior pattern^(2)^. In epidemiology, neural network models have been used to classify municipalities based on social vulnerability with high precision, highlighting those with extreme vulnerability from those that have the best social indicators^(18)^. During the COVID-19 pandemic, researchers used AI algorithms to define priority population groups for vaccination based on the highest risk of in-hospital death^(19)^. Specifically in nursing, there are few studies on the subject, highlighting the applicability of AI for assessing students’ emotions during clinical simulation, based on facial expressions^(20)^, and for detecting cases of sepsis^(21)^.

This study sought to integrate knowledge from AI with fundamental nursing knowledge, delving deeper into the analysis of predicting the execution time of an important routine nursing intervention aimed at promoting hygiene and comfort for critically ill patients^(22)^, the implementation of which impacts both patients and professionals. For patients, during bed bathing, there is a risk of infection, displacement of devices, falls from bed^(23)^, in addition to oxyhemodynamic changes^(10,12)^. For nursing professionals, less time spent showering means less work overload and less physical exhaustion^(8,24)^.

It is up to nurses to make decisions about when and how to provide bed baths, in addition to defining which strategies and resources were used, taking into account the use of validated protocols^(25)^. According to Professional Practice Law 7,498/86, nursing care directed to critical patients is considered a private activity for nurses^(26)^. However, it is observed that, in a large part of Brazilian ICUs, bed bathing is performed by the nursing technician, without any participation from a higher education professional^(8)^. Thus, based on the results of this work and, transcending theory, it is possible to critically analyze the scenario idealized by legislation and the real scenario experienced. It is necessary to reflect on the impact of using prediction of patient bed bath time for the nursing work process, whereas, based on prediction, it becomes possible to differentiate those who require more care time from those who require less time from the nursing team. From this reflection, it is possible to think about the diversity of conditions presented by critical patients and whether, perhaps, patients with less complexity can have their bath delegated in a regulatory manner to technical level professionals, maintaining the need for direct supervision of a nurse, which often already occurs in practice.

Furthermore, in clinical practice, it is recommended that bed baths be performed by more than one professional, in order to promote safer care, with less effort and greater agility^(8)^. However, there is a lack of professionals, which reinforces the importance of a tool that supports nurses in human resource management, based on the forecast of the time that each professional should allocate to carrying out baths for each patient, taking individual characteristics are taken into account. However, it should be noted that, even though the models present good predictive performance, they can generate inaccurate predictions. Therefore, for greater effectiveness, its use should not be conducted in isolation, but associated with clinical judgment carried out by professionals, which could translate into better care^(27)^.

### Study limitations

The results of this investigation must be interpreted with caution, as the data that gave rise to the model came from a single ICU, whose baths were always performed by two people. However, despite being a small sample, it does not present selection bias and is representative of the critically ill patient population.

### Contributions to nursing, health, or public policy

It is believed that the model built by AI analysis with good predictive capacity could create a useful tool to equip professionals in nursing care planning and execution during bed baths. By predicting the bathing time, nurses will be able to distribute care more equally among team members, minimizing work overload and the risks inherent to this intervention. It is expected that the possibility of predicting patient bed bath time before its execution can assist in the nursing team’s work process in ICUs, in order to contribute to saving time and improving care, directing in an equitable manner patients according to their care needs.

## CONCLUSIONS

It is concluded that, among the analytical methods used, the neural network model with a radial basis function presented the best predictive performance to estimate the bed bath execution time in critically ill patients, followed by perceptron neural network model 1. Thus, it is evident that AI can be useful for the construction of a tool that can guide nursing practice related to bed bathing, assisting professionals’ decision-making during the planning and execution of this intervention, with a view to improving the sizing of human resources and optimizing the work process.

## Supplementary Material

0034-7167-reben-77-01-e20230201-suppl01

## Data Availability

https://doi.org/10.48331/scielodata.HLY7DQ
