# Supplementary material for: Artificial intelligence to predict bed bath time in Intensive Care Units
Source: Rev Bras Enferm. 2024 Feb 26;77(1):e20230201. doi: 10.1590/0034-7167-2023-0201 (PMC10895787; doi:10.1590/0034-7167-2023-0201)
Supplement: 0034-7167-reben-77-01-e20230201-suppl01 [file 0034-7167-reben-77-01-e20230201-suppl01.pdf]

Idade	Sexo	Comorbidades	Sedativos	DVA	BIC	AVP	AVC
38.0	1.0	0.0	0.0	0.00	1.0	1.0	0.0
70.0	0.0	1.0	0.0	0.00	1.0	1.0	0.0
64.0	0.0	1.0	0.0	0.00	1.0	1.0	0.0
55.0	1.0	1.0	0.0	1.00	1.0	0.0	1.0
86.0	0.0	1.0	0.0	0.00	1.0	1.0	0.0
40.0	1.0	1.0	0.0	0.00	1.0	1.0	0.0
60.0	1.0	1.0	0.0	0.00	1.0	1.0	0.0
84.0	0.0	1.0	1.0	0.00	1.0	1.0	0.0
86.0	1.0	1.0	0.0	0.00	1.0	1.0	0.0
77.0	1.0	1.0	0.0	0.00	1.0	1.0	0.0
41.0	0.0	1.0	0.0	0.00	1.0	1.0	0.0
66.0	1.0	1.0	0.0	0.00	1.0	1.0	1.0
74.0	1.0	1.0	1.0	0.00	1.0	1.0	1.0
72.0	1.0	1.0	0.0	0.00	1.0	1.0	0.0
94.0	1.0	1.0	0.0	0.00	1.0	1.0	0.0
84.0	1.0	1.0	0.0	0.00	1.0	1.0	0.0
50.0	1.0	1.0	1.0	0.00	1.0	1.0	0.0
100.0	1.0	1.0	0.0	0.00	1.0	1.0	0.0
38.0	1.0	0.0	0.0	0.00	1.0	1.0	0.0
77.0	0.0	1.0	0.0	0.00	1.0	1.0	0.0
70.0	1.0	1.0	0.0	0.00	1.0	1.0	0.0
62.0	0.0	1.0	0.0	1.00	1.0	1.0	0.0
96.0	0.0	1.0	0.0	0.00	1.0	1.0	0.0
80.0	0.0	1.0	0.0	1.00	1.0	1.0	0.0
62.0	0.0	1.0	1.0	1.00	1.0	1.0	0.0
72.0	1.0	1.0	0.0	0.00	1.0	1.0	0.0
38.0	0.0	0.0	0.0	0.00	1.0	1.0	0.0
83.0	0.0	1.0	0.0	0.00	1.0	1.0	0.0
32.0	0.0	0.0	1.0	0.00	1.0	1.0	0.0
91.0	1.0	1.0	0.0	0.00	1.0	0.0	1.0
66.0	1.0	1.0	0.0	1.00	1.0	1.0	0.0
87.0	1.0	1.0	0.0	0.00	1.0	1.0	0.0
58.0	1.0	0.0	0.0	0.00	0.0	1.0	0.0
100.0	0.0	1.0	0.0	0.00	1.0	1.0	0.0
71.0	1.0	1.0	0.0	0.00	1.0	1.0	0.0
80.0	0.0	1.0	1.0	1.00	1.0	0.0	1.0
79.0	0.0	1.0	1.0	1.00	1.0	1.0	0.0
91.0	1.0	1.0	0.0	1.00	1.0	1.0	0.0
43.0	0.0	1.0	1.0	1.00	1.0	0.0	1.0
61.0	1.0	1.0	0.0	0.00	1.0	0.0	1.0
72.0	1.0	1.0	0.0	1.00	1.0	1.0	0.0
86.0	0.0	1.0	1.0	0.00	1.0	1.0	0.0
24.0	0.0	0.0	0.0	0.00	1.0	1.0	0.0
87.0	0.0	1.0	0.0	0.00	1.0	1.0	0.0
51.0	1.0	0.0	1.0	0.00	1.0	1.0	1.0
51.0	1.0	0.0	0.0	0.00	0.0	1.0	0.0
80.0	1.0	1.0	0.0	0.00	1.0	1.0	0.0
74.0	1.0	1.0	0.0	0.00	0.0	1.0	0.0
51.0	0.0	1.0	0.0	0.00	1.0	1.0	0.0

78.0	0.0	1.0	0.0	1.00	1.0	1.0	0.0

CVD	CNE	O2	TOT	MonitHemod	Dreno	Ostomia	Tempo_Execucao
0.0	0.0	0.0	0.0	0.0	0.0	0.0	30.25
0.0	1.0	0.0	0.0	0.0	0.0	0.0	34.25
0.0	0.0	0.0	0.0	0.0	1.0	0.0	29.29
1.0	1.0	1.0	1.0	1.0	0.0	0.0	40.20
0.0	0.0	1.0	0.0	0.0	0.0	0.0	28.12
0.0	0.0	0.0	0.0	0.0	0.0	0.0	24.59
1.0	0.0	0.0	0.0	0.0	0.0	0.0	26.42
1.0	0.0	1.0	1.0	0.0	0.0	1.0	28.19
1.0	0.0	1.0	0.0	0.0	0.0	0.0	32.59
1.0	1.0	0.0	0.0	0.0	0.0	0.0	28.47
1.0	1.0	0.0	0.0	1.0	1.0	0.0	29.59
0.0	0.0	1.0	0.0	0.0	0.0	0.0	24.31
1.0	1.0	1.0	1.0	1.0	0.0	0.0	34.35
0.0	0.0	0.0	0.0	0.0	0.0	0.0	30.13
1.0	0.0	1.0	0.0	0.0	0.0	0.0	28.52
0.0	0.0	1.0	0.0	0.0	0.0	0.0	27.10
0.0	1.0	1.0	1.0	0.0	0.0	0.0	30.39
1.0	0.0	1.0	0.0	0.0	0.0	0.0	27.01
1.0	1.0	1.0	0.0	0.0	1.0	0.0	33.39
0.0	0.0	0.0	0.0	0.0	0.0	0.0	22.50
1.0	1.0	1.0	1.0	0.0	0.0	0.0	26.30
0.0	0.0	1.0	0.0	0.0	0.0	0.0	27.38
1.0	0.0	1.0	0.0	0.0	0.0	0.0	35.10
0.0	0.0	0.0	0.0	0.0	0.0	0.0	19.44
1.0	1.0	1.0	1.0	0.0	0.0	0.0	22.50
1.0	0.0	0.0	0.0	0.0	0.0	0.0	19.26
0.0	0.0	0.0	0.0	0.0	0.0	0.0	22.13
0.0	0.0	0.0	0.0	0.0	0.0	0.0	22.52
0.0	1.0	1.0	1.0	0.0	0.0	0.0	25.43
1.0	1.0	1.0	1.0	0.0	0.0	0.0	29.52
0.0	0.0	0.0	0.0	0.0	0.0	0.0	19.39
1.0	0.0	1.0	0.0	0.0	0.0	0.0	29.15
0.0	0.0	0.0	0.0	0.0	0.0	0.0	20.39
1.0	0.0	1.0	0.0	0.0	0.0	0.0	23.10
0.0	0.0	0.0	0.0	0.0	0.0	0.0	17.47
1.0	1.0	1.0	1.0	1.0	0.0	0.0	33.14
1.0	1.0	1.0	1.0	0.0	0.0	0.0	25.08
1.0	1.0	1.0	0.0	0.0	0.0	0.0	29.52
1.0	1.0	1.0	1.0	0.0	0.0	0.0	27.20
1.0	1.0	1.0	0.0	0.0	1.0	0.0	26.25
1.0	0.0	1.0	0.0	0.0	1.0	0.0	28.34
1.0	1.0	1.0	1.0	0.0	0.0	0.0	23.19
0.0	1.0	0.0	0.0	0.0	0.0	0.0	23.39
1.0	0.0	1.0	0.0	0.0	0.0	0.0	23.20
1.0	0.0	0.0	0.0	0.0	1.0	1.0	24.52
0.0	0.0	0.0	0.0	0.0	0.0	0.0	18.47
1.0	1.0	1.0	0.0	0.0	0.0	0.0	24.41
0.0	0.0	0.0	0.0	0.0	0.0	0.0	21.32
0.0	0.0	0.0	0.0	0.0	0.0	0.0	20.11

0.0	0.0	0.0	0.0	0.0	0.0	0.0	25.57
